# Current Trends in Diagnosis and Early Monitoring of Oral Cavity Cancer: Techniques and Biomarkers

**DOI:** 10.3390/cancers18132088

**Published:** 2026-06-27

**Authors:** Karolina Maria Marczuk, Mateusz Bartosz Mamala, Alexandra Opalewski, Izabela Główka, Hanna Gerber, Andrzej Jaxa-Kwiatkowski

**Affiliations:** 1 Student Scientific Circle of Maxillofacial Surgery, Wroclaw Medical University, 50-345 Wroclaw, Poland; mateusz.mamala@student.umw.edu.pl (M.B.M.); alexandra.opalewski@student.umw.edu.pl (A.O.); izabela.glowka@student.umw.edu.pl (I.G.); 2 Clinical Department of Maxillofacial Surgery, Faculty of Dentistry, Wroclaw Medical University, 50-367 Wrocław, Poland; hanna.gerber@umw.edu.pl (H.G.); andrzej.jaxa-kwiatkowski@umw.edu.pl (A.J.-K.)

**Keywords:** oral squamous cell carcinoma, early detection, liquid biopsy, salivary biomarkers, optical imaging, brush cytology, artificial intelligence, biosensors

## Abstract

**Background/Objectives**: Oral cavity squamous cell carcinoma (OSCC) remains a major global health burden, with outcomes strongly dependent on stage at diagnosis. Although the oral cavity is directly accessible to clinical examination, many cases are still detected at advanced stages. This narrative review aimed to summarize current trends in OSCC diagnosis and early monitoring, with emphasis on non-invasive and minimally invasive techniques and biomarkers. **Methods**: A semi-systematic narrative literature search was conducted in PubMed/MEDLINE, Scopus, and Web of Science using predefined combinations of OSCC-, early-detection-, imaging-, cytology-, liquid-biopsy-, salivaomics-, artificial-intelligence-, and biosensor-related terms. English-language systematic reviews, meta-analyses, reviews of reviews, translational studies, and clinically relevant original articles were prioritized, with explicit attention to oral cavity-specific evidence and clearly identified extrapolation from broader head-and-neck or oropharyngeal cancer settings. **Results**: Conventional oral examination and histopathological assessment of biopsy specimens remain the diagnostic foundation. Adjunctive methods may support lesion triage, biopsy-site selection, risk stratification, and early monitoring, but cannot replace tissue diagnosis. Narrow-band imaging, optical coherence tomography, and molecular brush cytology appear particularly promising for specialist assessment and surveillance. Liquid biopsy and saliva-based biomarker platforms offer translational potential, particularly for repeatable monitoring, but clinical implementation is limited by methodological heterogeneity, pre-analytical variability, inconsistent thresholds, and insufficient external validation. Artificial intelligence and biosensor platforms remain promising but largely developmental. **Conclusions**: Progress in early OSCC diagnosis and monitoring will most likely depend on integrated diagnostic models combining clinical examination, adjunctive imaging, minimally invasive sampling, molecular biomarkers, and computational decision-support tools, validated in prospective multicenter studies.

## Simple Summary

Oral cavity cancer is often curable when found early, but many patients are still diagnosed after the disease has advanced. Because the mouth can be examined directly, better tools are needed to help clinicians recognize suspicious changes earlier, choose the right place for biopsy, and monitor patients after treatment. This review summarizes current and emerging methods for diagnosing and following oral cavity cancer, including special light-based imaging, brush-based cell sampling, saliva and blood tests, artificial intelligence, and biosensor technologies. We discuss which approaches are closest to clinical use and which still require stronger validation. The main message is that no single new test can replace careful clinical examination and tissue biopsy, but combining imaging, minimally invasive sampling, molecular markers, and computer-assisted analysis may improve early detection and patient monitoring in the future.

## 1. Introduction

### 1.1. Epidemiology and Clinical Importance of Early Detection in Oral Cavity Cancer

Oral cavity squamous cell carcinoma (OSCC) accounts for more than 90% of all malignant neoplasms arising in the oral cavity and remains a substantial global health burden. According to GLOBOCAN 2022 version 1.1 estimates, cancers of the lip and oral cavity accounted for 389,846 new cases and 188,438 deaths worldwide [1]. Although the conventional clinical profile of a patient with OSCC has been well described as that of an older male with a long-standing history of tobacco and alcohol exposure, the epidemiologic pattern appears to be shifting. OSCC is increasingly being diagnosed in individuals who do not fit this traditional risk profile. Population-level data indicate a rising incidence of OSCC among both men and women younger than 40 years of age [2]. This shift is clinically important because it broadens the population in whom early diagnostic vigilance is required.

In the United States, the tongue is the most frequent primary site of OSCC, accounting for 41.7% of cases, followed by the lip and floor of the mouth, each accounting for 16.5%; the gingiva, 10.6%; buccal mucosa, 6.7%; retromolar trigone, 5.6%; and hard palate, 2.3%. However, the epidemiology and site distribution of OSCC are not uniform and may differ substantially across geographic regions, including between individual countries and among subnational areas. These regional differences should therefore be considered when extrapolating such data to other populations [3].

Oral cavity cancer remains associated with substantial mortality, with approximately one-third of patients dying from any cause within five years and approximately one-fifth dying from the disease itself. These findings underscore the continued importance of advanced pathologic stage, particularly nodal involvement, as a principal determinant of fatal outcomes [4]. Accordingly, early-stage detection remains one of the most important opportunities to improve survival and reduce treatment-related morbidity. Treatment selection in OSCC depends on tumor stage, anatomic site, anticipated functional outcomes, and patient-related factors. In selected early-stage cases, both surgery and radiotherapy may offer curative treatment, whereas postoperative radiotherapy, with or without systemic therapy, is generally considered when adverse pathologic features are present [5,6].

Oral cancer is largely preventable, as tobacco use and alcohol consumption, recognized as the principal risk factors, are implicated in approximately 90% of cases and act synergistically in promoting carcinogenesis. Additional etiologic factors include ultraviolet (UV) radiation, particularly UVB in lip cancer; dental trauma; genomic susceptibility; Fanconi anemia; oral dysbiosis; and other less common contributors [7]. Selected viral associations have also been investigated, but human papillomavirus (HPV)-driven carcinogenesis is well established primarily for oropharyngeal squamous cell carcinoma, especially tonsillar and base-of-tongue tumors, whereas its etiologic contribution to true oral cavity SCC appears much smaller and remains controversial [8,9]. Therefore, HPV-related head-and-neck or oropharyngeal evidence should not be presented as directly applicable to oral cavity SCC unless the anatomical site is clearly specified. The atypical, non-smoking variant of OSCC appears to demonstrate a unique epigenetic profile that distinguishes it from the conventional tobacco-related form of the disease [2].

Taken together, these observations support the need for diagnostic strategies that extend beyond traditional risk-based clinical suspicion and enable earlier identification of biologically significant lesions. Although therapeutic strategies for oral cavity cancer have advanced substantially, survival outcomes remain closely linked to stage at diagnosis. Early detection is therefore clinically crucial, as it allows treatment at a localized stage, improves survival rates, reduces treatment morbidity, and enhances functional and quality-of-life outcomes [10].

### 1.2. Rationale for Non-Invasive and Adjunctive Diagnostics and Monitoring

Early identification of oral cancer and oral potentially malignant disorders (OPMDs) may enable less extensive treatment, improve prognosis, and enhance post-treatment quality of life [11,12]. In recent years, several non-invasive adjunctive systems, particularly fluorescence-based technologies, have been introduced to improve the detection and real-time assessment of suspicious lesions [13,14]. However, current evidence suggests that no single adjunctive method is sufficient as a stand-alone screening tool. Progress in early OSCC detection is more likely to depend on the integration of careful clinical examination with imaging-based adjuncts, cytology-based triage, and molecular biomarkers.

In this context, the present narrative review summarizes current diagnostic and early monitoring strategies for oral cavity squamous cell carcinoma, with particular emphasis on non-invasive and minimally invasive approaches, including optical adjuncts, cytology-based methods, liquid biopsy, salivaomics, artificial intelligence, and biosensor technologies. In addition to outlining the biological rationale for biomarker development, this review evaluates the current level of clinical applicability, major methodological limitations, and translational barriers that continue to hinder routine implementation. Particular attention is given to distinguishing tools intended for screening, lesion triage, risk stratification, and longitudinal surveillance. Accordingly, this review adopts a clear, clinically focused narrative approach, with transparent literature selection, a balanced interpretation of the evidence, and a critical synthesis of translational relevance.

The added value of this review is therefore integrative rather than duplicative. Recent systematic reviews have assessed individual subdomains such as conventional examination, optical adjuncts, cytology, liquid biopsy, salivary biomarkers, artificial intelligence, and biosensors. In contrast, the present review brings these domains together in a single oral cavity-focused framework, distinguishes screening, lesion triage, biopsy guidance, risk stratification, and surveillance use-cases, and proposes a stepwise diagnostic pathway that separates clinically established tools from adjunctive and still-investigational technologies. To keep this broad synthesis clinically focused, the review distinguishes four use-cases: screening of apparently healthy or high-risk individuals; lesion triage of visible or persistent abnormalities to decide on referral or biopsy; biopsy guidance to select the most suspicious site; and surveillance/early monitoring after OSCC treatment or during OPMD follow-up. Invasive OSCC, OPMD/dysplasia, and post-treatment recurrence are therefore interpreted as related but clinically distinct targets, because a method useful for OPMD risk enrichment may not be validated for early invasive OSCC detection or recurrence monitoring.

## 2. Materials and Methods

This is a semi-systematic narrative review designed to synthesize current and emerging approaches for the diagnosis and early monitoring of oral cavity squamous cell carcinoma. PubMed/MEDLINE, Scopus, and Web of Science were searched from 1 January 2014 to 7 April 2026; older landmark studies were included when they provided essential conceptual, methodological, or historical context. The final literature search was last updated on 7 April 2026. The search strategy used combinations of controlled and free-text terms, including: (“oral squamous cell carcinoma” OR “oral cavity cancer” OR “oral cancer” OR OSCC) AND (diagnosis OR “early detection” OR screening OR surveillance OR monitoring); (OSCC OR “oral squamous cell carcinoma”) AND (“liquid biopsy” OR ctDNA OR cfDNA OR “circulating tumor cells” OR exosomes OR “extracellular vesicles”); (OSCC OR “oral cancer”) AND (saliva OR salivary OR salivaomics OR miRNA OR metabolomics OR proteomics); (OSCC OR “oral potentially malignant disorders”) AND (autofluorescence OR chemiluminescence OR “narrow-band imaging” OR “optical coherence tomography” OR “high-resolution microendoscopy”); and (OSCC OR “oral cancer”) AND (“artificial intelligence” OR “machine learning” OR biosensor OR “point-of-care”). Reference lists of key systematic reviews and clinically relevant articles were also screened manually. Eligible sources included systematic reviews, meta-analyses, reviews of reviews, diagnostic-accuracy studies, prospective or retrospective clinical studies, translational biomarker studies, and technically relevant point-of-care or biosensor studies. Priority was given to oral cavity-specific evidence, histopathologically confirmed OSCC/OPMD cohorts, clinically relevant comparators, and studies reporting diagnostic performance, sample type, analytical platform, validation strategy, or translational readiness. Non-English articles, conference abstracts without sufficient methodological detail, studies focused only on treatment rather than diagnosis or monitoring, and studies not directly relevant to oral cavity cancer were excluded unless explicitly discussed as extrapolated evidence from broader head-and-neck or oropharyngeal SCC contexts. Because the objective was narrative synthesis rather than quantitative pooling, no meta-analysis was performed, and no formal risk-of-bias scoring was applied across all included studies. However, the interpretation of diagnostic performance estimates considered study design, sample size, comparator group, external validation, pre-analytical standardization, and whether the evidence was oral cavity-specific or extrapolated from broader HNSCC/OPSCC literature. The final reference set comprised 82 sources. The search was performed iteratively across overlapping databases rather than as a registered PRISMA systematic review. The reasons for exclusion were predefined and included duplicate or clearly irrelevant records, non-English language, conference abstracts without sufficient methodological detail, treatment-only focus, absence of oral-cavity relevance, lack of histopathologic confirmation where diagnostic accuracy was claimed, and evidence derived from broader HNSCC/OPSCC settings without clear applicability to oral cavity disease. Studies were narratively grouped by modality and intended clinical function: conventional examination and biopsy, optical adjuncts, cytology-based methods, liquid biopsy, salivaomics, artificial intelligence, biosensor technologies, methodological limitations, and future directions. Evidence maturity was assigned descriptively using prespecified criteria. High indicates a reference standard, guideline-supported method, or broadly established clinical practice. Moderate to high indicates consistent systematic-review/meta-analytic or larger clinical evidence, but with implementation constraints or need for standardized interpretation. Moderate indicates multiple clinical studies or reviews with relevant heterogeneity, limited external validation, or uncertain stand-alone utility. Emerging indicates promising early clinical or translational evidence, commonly from small, selected, case–control, or single-center cohorts. Developmental indicates proof-of-concept, technical, preclinical, or early pilot evidence without robust prospective clinical validation.

Diagnostic performance values were interpreted according to intended clinical use, study design, population, comparator, and reference standard. Values from case–control or specialist-center studies were treated as potentially optimistic for unselected dental practice or population-level screening. Therefore, sensitivity, specificity, and AUC values in this review are used to support modality-specific interpretation rather than direct ranking across screening, triage, biopsy guidance, and surveillance contexts.

## 3. Pathophysiological Basis for Biomarker Selection

### 3.1. Tumor Biology of Oral Squamous Cell Carcinoma: Common Mutations and Pathways

Genetic alterations that contribute to oral carcinogenesis require continued investigation to enable a deeper and more precise characterization of tumor biology. Cytogenetic methods can play an important role in identifying and profiling these abnormalities [15]. OSCC is driven by recurrent genetic alterations that disrupt genome integrity, cell-cycle control, and growth-factor signaling. Across sequencing studies, the most consistently mutated genes include *TP53*, often the dominant event in HPV-negative head and neck cancers, as well as *CDKN2A*, *NOTCH1*, *FAT1*, and *PIK3CA*. These alterations reflect the convergent disruption of tumor suppression, differentiation programs, and oncogenic signaling pathways [16].

Somatic copy-number alterations (SCNAs) represent a common feature of OSCC and may affect oncogenes, tumor suppressor genes, passenger loci, and potential co-driver loci [17]. In one multiplex ligation-dependent probe amplification-based study of 33 OSCC cases, clinically relevant copy-number changes were identified across 30 chromosome 8 loci spanning 8p23–8q24, including alterations involving *FGFR1*, *PRKDC*, *EIF3E/EIF3H*, *MYC*, *PTK2*, *PTP4A3,* and *RECQL4*. These findings support the biological relevance of chromosome 8 instability in oral carcinogenesis [18].

Tobacco and alcohol exposure, both established drivers of genomic disruption, are strongly linked to OSCC incidence [19,20]. These exposures may contribute to alterations affecting key tumor suppressor and oncogenic pathways, including *TP53* dysfunction, *MYC* deregulation, *RAS*-associated signaling, and carcinogen-metabolizing systems such as *GSTM1*, thereby promoting malignant transformation and tumor progression [17,20].

### 3.2. How Tumor Shedding and Microenvironment Changes Create Detectable Signals

OSCC-driven microenvironment remodeling, including immune-inflammatory shifts, metabolic rewiring, extracellular matrix disruption, and microbiome dysbiosis, generates measurable changes in saliva, blood, and imaging-derived tissue phenotypes. These changes produce multimodal signals that machine learning approaches may integrate for diagnosis and prognostic stratification [21].

OSCC-associated DNA methylation signatures represent attractive candidates for early detection because they can be interrogated across multiple biologically distinct sampling matrices, including saliva and oral rinse, oral brushing specimens, and blood-derived cell-free DNA. In this context, saliva and oral rinse may capture locally shed epithelial and cell-free DNA signals, potentially reflecting both tumor-derived and field-level epigenetic alterations. Oral brushing, by contrast, provides a cell-enriched sample obtained in close proximity to focal lesions, and blood-based assays typically interrogate lower-abundance circulating tumor DNA [22].

Recent evidence supports the feasibility of methylation analysis across all of these specimen types. However, systematic methodological appraisal using QUADAS-2 has shown that diagnostic performance estimates may be inflated by case–control sampling, non-standardized thresholds, and limited blinding [23]. Accordingly, the clinical translation of methylation-based testing as a reliable screening or risk-stratification tool will require prospectively designed, head-to-head multicenter studies with harmonized pre-analytical workflows, predefined analytical cut-offs, and explicit control for confounding introduced by inflammation and OPMDs [24].

As discussed in more detail in Section 7.4, recent salivary miRNA research provides an illustrative example of how locally shed molecular signals may support early OSCC detection and risk stratification. The strong diagnostic separation achieved by an eight-salivary microRNA (miRNA) panel supports the concept that OSCC and high-risk OPMDs generate detectable signals through tumor shedding into saliva, particularly via exosome-associated miRNAs and exfoliated epithelial cells. The accompanying risk score further suggests that salivary miRNA patterns may track early microenvironmental remodeling in OPMDs, enabling minimally invasive identification of lesions at increased risk of progression to cancer [25].

Integrated transcriptomic and network analyses indicate that OSCC generates detectable biomarker signals through the combination of tumor-intrinsic regulatory rewiring, including competing endogenous RNA networks, and microenvironment-driven pathways, particularly cytokine-receptor signaling and NF-κB-mediated inflammation. Hub nodes such as *IGF2BP1*, *CLDN6,* and *HLA-G* point to coordinated changes in epithelial barrier function, adhesion, and immune modulation. These changes may be reflected in shed RNA species, including exosomal or secreted transcripts, thereby supporting minimally invasive detection and risk stratification [26].

## 4. Conventional Diagnostic Baseline: Visual Inspection, Biopsy, and Limitations of Current Pathways

In a meta-analysis of patients with clinically evident oral lesions, conventional oral examination showed summary estimates of 71% sensitivity and 85% specificity for detecting dysplastic and/or malignant disease [11]. A separate meta-analysis that included oral cavity and oropharyngeal precancerous/cancerous lesions reported lower pooled estimates for clinical visualization (63% sensitivity and 78% specificity) [27]. These figures should not be interpreted as contradictory point estimates for an identical test population; they reflect differences in included anatomical sites, lesion spectra, comparators, and study eligibility criteria. Taken together, the findings underscore that a clinical appearance of “benign” cannot reliably exclude clinically significant pathology.

Histopathologic assessment of tissue biopsy remains the diagnostic gold standard for suspicious oral lesions [28,29]. Scalpel and punch biopsies remain the most widely used approaches in routine practice. By contrast, the use of laser- or electrosurgery-based techniques should be used with caution in lesions suspected of dysplasia or malignancy, because thermal artifact may compromise tissue readability and margin interpretation, particularly when technical parameters are suboptimal [30].

An additional limitation of biopsy-based diagnosis is sampling error, as different areas of the same lesion may demonstrate varying degrees of dysplasia or even early invasion. Consequently, careful selection of the most representative biopsy site, adequate biopsy depth, and multiple biopsies when appropriate are important for improving diagnostic accuracy [31]. The systematic review by Yan et al. highlights the broad range of specimen types currently used in oral cancer detection. These include conventional invasive tissue samples, such as primary tumor or lesion tissue, and, where relevant, lymph node tissue, as well as less invasive liquid-biopsy specimens, including saliva, blood, and urine. Such specimens may support downstream molecular analyses alongside standard histopathology [32]. In cases of suspected malignant cervical lymphadenopathy, adult neck-mass guidelines recommend fine-needle aspiration (FNA) rather than open biopsy when the diagnosis remains uncertain [33].

Despite the accessibility of the oral cavity to direct clinical examination, diagnostic delay remains a substantial problem. One review estimated that approximately two-thirds of patients with oral cancer are still diagnosed at an advanced stage, defined as stage III or IV disease [34]. Adjunctive approaches, including cytology-based tests, vital staining, autofluorescence, and tissue reflectance methods, may assist in lesion triage and in identifying patients who warrant referral or biopsy. However, an ADA-commissioned systematic review emphasized that the supporting evidence remains of low or very low quality and cautioned that false-positive findings represent a major limitation of these modalities [35]. In practical terms, the same diagnostic tool may have different value depending on the clinical question. In screening, false positives and referral burden are central; in lesion triage, the main issue is whether a tool accelerates appropriate biopsy or specialist referral; in biopsy guidance, the relevant outcome is more accurate sampling of the highest-risk region; and in surveillance, repeatability and early recurrence detection must be balanced against cost, anxiety, and false alarms.

## 5. Optical and Imaging Adjuncts for Early Detection

Conventional white-light examination remains the foundation of initial clinical assessment and opportunistic screening for oral mucosal lesions. Optical adjuncts are primarily intended to improve lesion triage, guide biopsy site selection, and support the follow-up of OPMDs, rather than replace histopathology [12,13].

In a meta-analysis comparing light-based adjuncts with clinical visualization, pooled diagnostic performance was moderate for conventional clinical examination, with a sensitivity of 63% and specificity of 78%. Autofluorescence-based tests showed higher sensitivity but still imperfect specificity, with pooled estimates of 86% and 72%, respectively, whereas chemiluminescence showed comparatively weaker specificity, with a sensitivity 67% and a specificity of 48% [27]. As noted above, these values differ from the 71%/85% estimates reported by Essat et al. [11] because the meta-analyses used different inclusion criteria and comparator frameworks. These findings underscore that light-based adjuncts may increase lesion detection, but often at the cost of higher false-positive rates [27].

Autofluorescence devices, such as VELscope and related systems, are based on the observation that normal mucosa emits pale green autofluorescence, whereas abnormal tissue may show reduced or altered fluorescence. Pooled evidence suggests that these systems function mainly as sensitivity-oriented adjuncts rather than definitive rule-in tests [27,36]. At the same time, multiple reviews emphasize that benign conditions, particularly inflammation and other non-neoplastic mucosal changes, may also alter fluorescence patterns, thereby reducing specificity. This limitation is especially problematic in low-prevalence screening settings [37,38].

Chemiluminescence, classically performed after a 1% acetic-acid rinse using blue-white light at approximately 490–510 nm, may enhance the visual conspicuity of mucosal abnormalities. However, evidence syntheses continue to show variable diagnostic performance and limited strong support for broad screening use, consistent with the relatively low pooled specificity reported in meta-analyses [13,27,36].

Narrow-band imaging (NBI) enhances visualization of the mucosal and submucosal microvasculature through narrow-band blue and green illumination. It is therefore well-suited conceptually for the detection of premalignant and malignant lesions characterized by abnormal vascular architecture. A dedicated meta-analysis in OPMD/OSCC reported, for a commonly used intrapapillary capillary loop threshold, a pooled sensitivity of 87% (95% CI, 67–96%), specificity of 83% (95% CI, 56–95%), and an AUC of 0.92 (95% CI, 0.89–0.94), suggesting comparatively strong diagnostic performance among currently available optical adjuncts [37,39].

High-resolution endoscopic approaches may complement wide-field flagging methods by enabling more detailed assessment of suspicious areas. In small specialist studies, contact endoscopy has been reported to have a sensitivity of approximately 80%, specificity up to 100%, and accuracy of approximately 93% for malignancy; however, the apparent 100% specificity should not be overinterpreted because it derives from limited, selected cohorts rather than broad real-world validation. High-resolution microendoscopy (HRME) achieved a sensitivity of 85–90% and a specificity of 80–85% by expert interpretation, with automated classification yielding sensitivity and specificity of 81% and 77%, respectively, in head and neck/oral neoplasia settings [37,40].

Among newer modalities, optical coherence tomography (OCT) offers non-invasive, cross-sectional, near-histologic visualization of epithelial and subepithelial architecture. A recent systematic review summarized clinician-interpreted sensitivities of approximately 75–92% and specificities of 71–93%, with even higher figures reported in selected algorithm-assisted studies, including sensitivity and specificity up to 98.5% and 99%, respectively, in one AI-supported approach [41]. More recent critical review data also support the view that OCT is among the most promising emerging modalities for real-time assessment of OPMDs/OSCC and for selected surgical-margin applications, although standardization and broader validation remain necessary before widespread clinical adoption [12,41].

For surveillance-oriented and precision-guided applications rather than first-line community screening, autofluorescence-guided excision has been reported in a specialist/single-study setting to yield a higher rate of tumor-free margins than standard excision, at 97% versus 73%. This finding is consistent with broader head and neck optical imaging literature emphasizing the potential role of optical adjuncts in real-time delineation and surgical support, but the precision of these estimates should not be taken to imply definitive generalizability without larger multicenter validation [39,42].

Finally, narrow-band/LED multispectral imaging is emerging as a data-rich extension of optical assessment. At least one multispectral tissue-classification study reported sensitivity of 85.3% and specificity of 70.8%; however, this literature remains largely developmental and should currently be regarded as promising but insufficiently validated for routine clinical implementation or population-level screening [36,43].

Taken together, currently available optical adjuncts appear most useful as lesion triage and biopsy-guidance tools, particularly in specialist settings and in the follow-up of OPMDs. Among these modalities, NBI currently appears to offer the most favorable balance between sensitivity and specificity for triage. In contrast, chemiluminescence and autofluorescence are more strongly limited by false-positive findings, especially in inflammatory or otherwise benign mucosal conditions. In translational terms, NBI and OCT appear to have the greatest clinical readiness, while multispectral and other AI-integrated optical platforms remain promising but still largely developmental [12,13]. From an implementation perspective, NBI and OCT are more plausible for specialist oral medicine, maxillofacial, otolaryngology, or oncology settings than for routine general dental screening. Their use requires equipment, operator training, standardized interpretation, chairside time, and a referral pathway that leads rapidly to biopsy when concern persists. In general dental practice, their value is likely to remain limited unless embedded within clear referral protocols and local specialist access.

## 6. Cytology and Point-of-Care Tissue Tests

Brush-based oral cytology provides a minimally invasive means of sampling the oral epithelium and may support the early assessment and triage of OPMDs. However, histopathology remains the diagnostic reference standard. In a PRISMA-DTA systematic review and meta-analysis that included 53 studies and 13,249 patients, oral cytology demonstrated a pooled sensitivity of 91.4% (95% CI, 87.8–94.1%) and specificity of 96.0% (95% CI 93.7–97.5%) for the detection of OSCC/OPMD when compared with histopathology. These findings support its role as a clinically relevant adjunct in early lesion assessment [44].

Liquid-based cytology is often regarded as a higher-performing variant of exfoliative cytology. Comparative clinical data indicate improved diagnostic characteristics over conventional smears. For example, in a 251-case series, liquid-based cytology showed a sensitivity of 79.4% versus 76.7% and a specificity of 85.1% versus 69.2% when “OHSIL/SCC” categories were considered cytology-positive [45]. Nevertheless, even liquid-based cytology remains subject to clinically relevant misclassification. In the same study, the false-positive rate was 14.9%, and the false-negative rate was 20.6%, indicating that cytologic findings should be interpreted in conjunction with the clinical context rather than used as stand-alone rule-in or rule-out tests [45].

Toluidine blue is a simple outpatient adjunct that preferentially stains acidic tissue components, including DNA and RNA, and has been used to support biopsy site selection and the follow-up of premalignant lesions. Its limitations are clinically relevant. Inflammatory or ulcerated lesions may retain dye and generate false-positive findings, whereas hyperkeratotic lesions may reduce dye penetration and contribute to false-negative results [46]. A systematic review and meta-analysis concluded that toluidine blue does not have sufficient diagnostic accuracy to be used in isolation and should instead be combined with other adjunctive approaches in the diagnostic workup [47].

Point-of-care approaches are increasingly extending beyond morphology alone by integrating brush sampling with automated imaging and molecular readouts. A multicenter validation study of a cytology-on-a-chip platform reported high automated cell-phenotype classification performance. In that study, clinical algorithms achieved an AUC of 0.81 for benign versus mild dysplasia and 0.95 for benign versus malignancy, supporting the feasibility of more rapid and potentially scalable lesion assessment across different care settings [48].

Adjunctive molecular testing of brush samples, particularly DNA ploidy analysis and image cytometry, has also shown strong performance for identifying high-grade disease. Reported findings include a sensitivity of 93.5% for severe dysplasia, carcinoma in situ, and OSCC in a large prospective cohort. In a DNA-ploidy oral cytology algorithm study, sensitivity was reported as 96–97% for detecting high-grade lesions. Meta-analytic evidence further suggests that cytology performs better when combined with DNA analysis than when used alone [49]. Recent point-of-care reviews likewise support the translational potential of integrated cytology-plus-biomarker platforms, particularly in settings where rapid, minimally invasive, and scalable lesion assessment is desirable [50].

Overall, brush cytology, toluidine blue, and point-of-care molecular kits are best regarded as adjunctive triage and surveillance tools. These approaches may help prioritize lesions that require prompt scalpel biopsy or closer follow-up, but they should not delay definitive histopathologic diagnosis when clinical suspicion persists [44]. From a practical standpoint, brush cytology, particularly when combined with adjunctive molecular analysis, appears to have the greatest translational readiness among cytology-based approaches for lesion triage. By contrast, toluidine blue is more clearly constrained by false-positive findings related to inflammation and ulceration, limiting its value as a stand-alone test. Taken together, cytology-plus-molecular platforms appear to offer the strongest near-term clinical potential, especially in settings where minimally invasive assessment and repeat sampling are desirable [49,50]. Brush cytology may be more feasible than advanced optical systems in broader dental settings, but only when results are linked to clear management rules. A negative or low-risk result should not reassure clinicians when a lesion is persistent, indurated, ulcerated, erythroplakic, or otherwise clinically suspicious. Molecularly enhanced brush testing should therefore be considered a triage or risk-enrichment tool for selected lesions and OPMDs, not a substitute for biopsy or a population-level screening test.

## 7. Liquid Biopsy Approaches

Liquid biopsy refers to the analysis of tumor-derived materials released into body fluids rather than directly sampled from tissue [51]. In oral oncology, this approach has become increasingly relevant because OSCC may shed biologically informative material into locally accessible fluids, such as saliva and oral rinse, as well as into systemic compartments, such as plasma [52].

From a clinical perspective, the major appeal of liquid biopsy lies in its minimally invasive and repeatable nature. These features make it particularly attractive not only for early detection in high-risk patients but also for longitudinal monitoring of disease burden, treatment response, minimal residual disease, and early recurrence.

Importantly, however, liquid biopsy should currently be viewed as an adjunct to, rather than a replacement for, conventional histopathologic diagnosis. Recent reviews consistently emphasize that its greatest present value lies in complementary risk stratification and surveillance. Routine diagnostic integration will still require assay harmonization, prospective validation, and clinically standardized thresholds [51,52,53]. For clarity, liquid-biopsy evidence should be interpreted separately according to the target condition and intended use. Salivary or oral-rinse assays may be most relevant for local OPMD/OSCC signal capture and lesion triage, whereas plasma ctDNA and CTCs currently have stronger conceptual value for systemic tumor burden, prognosis, minimal residual disease, and recurrence surveillance. Consequently, high sensitivity, specificity, or AUC values from selected biomarker cohorts should not be interpreted as evidence that liquid biopsy can independently diagnose OSCC in routine practice without histopathologic confirmation.

### 7.1. Biological Basis

The biological rationale for liquid biopsy in OSCC is strong because malignant and premalignant lesions release a broad spectrum of analytes during tumor growth, local invasion, inflammation, hypoxia, and microenvironmental remodeling. These analytes include circulating tumor DNA (ctDNA), total cell-free DNA (cfDNA), circulating tumor cells (CTCs), miRNAs, messenger RNAs, proteins, metabolites, and extracellular vesicles (EVs), particularly exosomes. Their release may occur through apoptosis, necrosis, active secretion, or epithelial shedding into saliva and oral rinse [54].

This biological diversity is clinically relevant because different analytes provide distinct types of information. ctDNA reflects tumor-specific genomic and epigenomic aberrations, whereas CTCs offer insight into dissemination and metastatic competence. Salivary RNAs and EV cargo may capture locally enriched signals from oral lesions, including changes associated with field cancerization. For this reason, recent literature increasingly supports multi-analyte and multi-matrix strategies, rather than dependence on a single biomarker class, as the most realistic path toward clinically meaningful liquid-biopsy applications in OSCC [51,54].

### 7.2. Circulating Tumor DNA and Cell-Free DNA

ctDNA is the tumor-derived fraction of total cfDNA and carries somatic mutations, copy-number alterations, and methylation abnormalities that mirror the molecular profile of the primary lesion. In OSCC, ctDNA has attracted particular interest due to its high molecular specificity. However, its clinical sensitivity in early-stage disease remains constrained by low tumor fraction, especially in plasma. This limitation has driven interest in oral rinse and saliva as potentially more informative local substrates [55].

In a 2025 study, Chen et al. demonstrated that ctDNA was detectable in 94.3% of oral-rinse samples and 80.5% of plasma samples from patients with HPV-negative OSCC. Integrated oral-rinse and plasma analysis improved mutation profiling and recurrence prediction. The same study reported that longitudinal ctDNA monitoring detected recurrence approximately four months before clinical manifestation, supporting its possible role in early surveillance. Nevertheless, the authors also make clear that broader translation depends on standardized sample processing, robust assay reproducibility, and validation in routine clinical cohorts [52].

Beyond mutation detection alone, cfDNA-based approaches are increasingly being explored for fragmentomics and methylation profiling. These approaches may be particularly valuable when tumor DNA abundance is low. This is an important direction because epigenetic disruption often occurs early in oral carcinogenesis and may therefore provide diagnostically useful signals even when sequence-based mutation analysis is less sensitive [56].

At the same time, methodological concerns remain substantial. Recent reviews of DNA-based liquid biopsy in OSCC repeatedly note the effects of pre-analytical variability, limited cohort sizes, inconsistent cut-offs, and the need to distinguish true tumor-derived signals from background noise and non-tumor sources of cfDNA. Therefore, ctDNA and cfDNA are best presented as high-potential tools for molecular stratification and surveillance rather than as established stand-alone screening tests [51,56].

### 7.3. Circulating Tumor Cells

CTCs are intact malignant cells that have detached from the primary tumor and entered the bloodstream. In theory, they are highly informative because they preserve cellular morphology and phenotype, thereby enabling genotypic and phenotypic characterization that cannot be obtained from fragmented nucleic acids alone.

In OSCC, CTCs are biologically linked to epithelial-to-mesenchymal transition (EMT), invasive behavior, metastatic spread, and, in some models, even tumor self-seeding. Despite this conceptual relevance, their diagnostic role in routine practice for oral cancer remains limited. The main difficulty is that CTCs are rare in peripheral blood, especially in earlier-stage disease. In addition, capture technologies differ in sensitivity depending on whether they rely on epithelial markers, size-based separation, or other enrichment strategies. As a result, cells undergoing EMT may be underdetected by conventional epithelial-marker-based assays. Current evidence, therefore, positions CTCs more strongly as prognostic and disease-monitoring biomarkers than as first-line early detection tools in OSCC [51,57].

### 7.4. Noncoding RNAs and Extracellular Vesicles

Noncoding RNAs, especially miRNAs, have emerged as one of the most promising classes of salivary biomarkers in oral oncology because of their relative stability, functional relevance, and detectability in noninvasive samples. Salivary miRNAs participate in complex regulatory networks affecting proliferation, apoptosis, invasion, immune modulation, and treatment resistance. Their diagnostic appeal is further strengthened by the fact that many miRNAs are transported in EVs, particularly exosomes, which protect nucleic acids from enzymatic degradation and reflect active tumor-microenvironment communication [58,59].

In a 2024 study, Balakittnen et al. validated multiple saliva-based miRNA models in oral cancer (OC; n = 50), OPMD (n = 52), and control (n = 60) cohorts. An eight-miRNA signature discriminated OC from controls with an AUC of 0.954, sensitivity of 86%, and specificity of 90%. In the same study, a distinct four-miRNA subpanel discriminated OC from OPMDs with an AUC of 0.9115, sensitivity of 90%, and specificity of 82.7%, while another four-miRNA model separated OPMDs from controls with an AUC of 0.807. The probability score also differentiated high-grade dysplasia from controls and from early cancer in a biologically meaningful gradient. These findings suggest that salivary miRNA signatures may have utility not only in the detection of oral cancer but also in the stratification of malignant transformation risk across the OPMD-OSCC spectrum [25].

The role of extracellular vesicles warrants separate emphasis, as exosomes may carry RNA, DNA, proteins, lipids, and signaling molecules that more comprehensively reflect tumor biology than isolated biomarkers. Recent systematic reviews of salivary exosomal miRNAs in head and neck squamous cell carcinoma have concluded that these markers are promising for both diagnosis and prognosis. However, these reviews have also highlighted persistent limitations, including small cohorts, inconsistent isolation methods, and limited external validation, and the fact that some evidence is derived from broader HNSCC cohorts rather than oral cavity-specific OSCC populations. Thus, although salivary miRNAs and exosomal cargo are among the most compelling candidates in liquid-biopsy research, their current role in clinical practice is best described as emerging rather than established [60,61].

### 7.5. Analytical Platforms

The diagnostic utility of liquid biopsy is strongly dependent on the analytical platform used for biomarker detection. Droplet digital PCR (ddPCR) is particularly effective for highly sensitive, absolute quantification of predefined low-abundance mutations or methylation targets. In contrast, targeted next-generation sequencing (NGS) allows broader molecular characterization, including detection of somatic mutations and copy-number alterations. Methylation-based techniques such as methylation-specific PCR and quantitative methylation assays are of particular interest in early detection studies because they target epigenetic abnormalities that may arise early in oral carcinogenesis.

Because these methodologies interrogate distinct dimensions of tumor biology and differ in terms of analytical scope, sensitivity, cost, and clinical feasibility, they should be regarded as complementary rather than interchangeable. Consequently, multi-platform strategies may enhance diagnostic performance by providing a more comprehensive assessment of tumor-derived molecular alterations [51,52]. Clinical interpretation also depends on the sampling matrix and intended scenario. Saliva and oral rinse may be more relevant for local lesion-associated shedding and OPMD/OSCC triage, whereas plasma ctDNA may be more informative for systemic tumor burden, minimal residual disease, or recurrence surveillance. At present, neither matrix has sufficiently standardized evidence for stand-alone population screening or routine general dental use.

### 7.6. Comparative Overview

Taken together, current evidence suggests that the different classes of liquid-biopsy analytes should be viewed as complementary rather than competitive. ctDNA and cfDNA are strongest for molecular characterization and recurrence monitoring. CTCs are most informative for dissemination biology and metastatic potential, whereas salivary RNAs and EV-associated analytes may be especially useful for local lesion detection and dynamic risk assessment.

Accordingly, the most plausible future direction is not a single universal liquid-biopsy marker, but integrated panels that combine analytes with different biological origins and clinical functions [51,52]. The comparative characteristics of the major liquid-biopsy analytes discussed in this review are summarized in Table 1.

## 8. Salivaomic Approaches

Saliva is one of the most attractive biofluids for OSCC biomarker research because it can be collected non-invasively, repeatedly, and inexpensively, while remaining in direct contact with the primary lesion and the surrounding altered mucosal field [63]. This anatomic proximity is highly relevant to early detection, as it increases the likelihood of capturing tumor-derived cells, nucleic acids, proteins, metabolites, and EVs before these signals become consistently detectable in systemic circulation.

Contemporary reviews, therefore, increasingly position saliva not merely as a convenient specimen but as a biologically informative matrix with particular value in oral carcinogenesis. Even so, the literature remains clear that salivary diagnostics are still investigational, and histopathologic examination remains the reference standard for definitive diagnosis [54,63].

### 8.1. Tumor-Derived Proteins and Metabolites

Salivary proteins have been studied for many years in OSCC, particularly inflammatory cytokines and matrix-remodeling molecules such as IL-1β, IL-6, IL-8, TNF-α, MMP-2, MMP-9, LDH, and CYFRA 21-1. In a 2024 exploratory systematic review, Bastías et al. reported that TNF-α, IL-1β, IL-6, IL-8, LDH, and MMP-9 were among the most frequently assessed salivary molecules and were repeatedly identified as promising candidates. However, the authors also emphasized that larger, better-standardized studies are still required before these biomarkers can be translated into clinical use. This caveat is important because protein biomarkers often perform well in small discovery cohorts but remain vulnerable to confounding by oral inflammation, periodontal disease, and interindividual variability [63].

More recently, metabolomics has attracted substantial interest because it captures low-molecular-weight compounds that may reflect tumor metabolism more directly than upstream genomic or transcriptomic alterations. A 2024 systematic review by Antonelli et al. [64] found that more than 100 salivary metabolites had been investigated in association with histologically confirmed OSCC. All included studies reported at least one statistically significant association, although most studies were of intermediate rather than high methodological quality. These findings suggest genuine biological promise but also highlight limitations in the current evidence base.

Notably, a 2026 targeted metabolomics study by Panneerselvam et al. collected unstimulated saliva from healthy controls (n = 15), oral leukoplakia patients (n = 15), early-stage OSCC patients (n = 28), and advanced-stage OSCC patients (n = 46). The study identified salivary N1-acetylspermidine and N1-acetylspermine as promising biomarkers for non-invasive OSCC detection and staging. Both metabolites showed progressive elevation from oral leukoplakia to advanced OSCC, with reported AUCs of 0.87 and 0.81, respectively; however, these findings should be interpreted as promising single-study evidence requiring independent validation [65].

### 8.2. Integration of Salivary and Blood-Based Approaches

An increasingly persuasive concept in the literature is that saliva and blood should not be framed as competing liquid-biopsy sources, but rather as biologically complementary matrices. Saliva may be more informative for local tumor activity, epithelial shedding, and early mucosal change, whereas plasma may better reflect systemic dissemination, total tumor burden, and recurrence dynamics beyond the oral cavity.

This complementarity is supported by a 2025 dual-source study by Chen et al. and by more recent transcriptomic comparisons suggesting that saliva may outperform plasma for some cfRNA signals in OSCC. Taken together, these findings indicate that saliva and plasma may provide distinct yet complementary molecular information, with the optimal matrix depending on the specific clinical context and diagnostic objective [52,66].

### 8.3. Pre-Analytical Considerations

Pre-analytical variability remains a principal challenge in developing reliable salivary biomarkers for OSCC. The diagnostic performance of saliva-based assays may be substantially influenced by whether samples are collected under stimulated or unstimulated conditions, as well as by the time of collection, recent food or tobacco exposure, hydration status, oral hygiene, periodontal inflammation, microbial contamination, storage conditions, and repeated freeze–thaw cycles. These factors can significantly alter biomarker concentration and composition, thereby contributing to inter-study heterogeneity and limiting reproducibility across independent cohorts [63].

**Figure 1:**
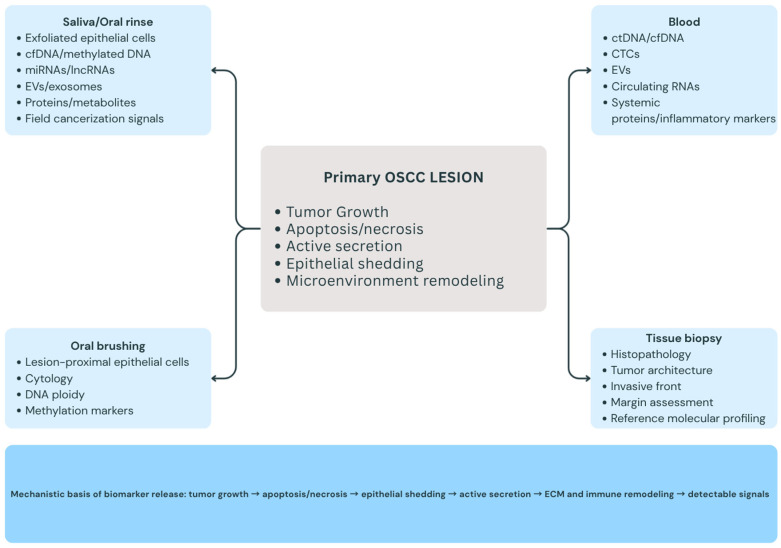
Biological origins of detectable signals in OSCC. Original figure created by the authors.

Consequently, the lack of standardized protocols for sample collection, processing, storage, and normalization remains a major obstacle to the clinical translation of salivary diagnostics. Future salivaomics studies should therefore place greater emphasis on rigorous pre-analytical control and transparent methodological reporting to improve comparability, reproducibility, and clinical applicability [67]. To integrate the diagnostic approaches discussed throughout the review, Table 2 provides a comparative overview of current and emerging modalities for early OSCC assessment, whereas Figure 1 summarizes the main biological sources of detectable OSCC-related signals across saliva or oral rinse, oral brushing specimens, blood, and tissue biopsy.

Table 2 should be read as a clinical-readiness summary rather than a formal grading-of-recommendations table. The categories reflect available evidence type, standardization, external validation, routine availability, and plausible role in screening, lesion triage, biopsy guidance, or surveillance. They do not imply direct comparability of performance values across modalities, because populations, reference standards, and study designs differ substantially.

## 9. Methodological Limitations and Translational Barriers

Despite the growing number of studies investigating novel diagnostic tools and biomarkers for early OSCC detection, the current evidence base remains limited by several recurring methodological and translational challenges that cut across imaging, cytology, liquid biopsy, salivaomics, AI, and biosensor studies. The cross-cutting limitations are: limited reproducibility, insufficient external validation, substantial interstudy heterogeneity, and uncertain performance in clinically representative populations.

A major limitation of the existing literature is the predominance of small, single-center, case–control studies. Such designs reduce statistical power, limit subgroup analyses, and may overestimate diagnostic accuracy, particularly when OSCC is compared with healthy controls rather than with clinically relevant comparator lesions such as OPMDs or inflammatory oral conditions. As a result, findings that appear promising in narrowly selected cohorts may not be reproducible in broader and more heterogeneous clinical populations. Larger prospective multicenter studies with clinically representative cohorts are therefore needed to establish the true diagnostic value of emerging biomarkers and adjunctive technologies [25,51,63,64,76].

Another major challenge is the limited translation of promising methods into routine practice. High analytical accuracy in controlled research settings does not necessarily translate into clinical utility, particularly when technologies require specialized equipment, advanced laboratory infrastructure, or computational support. This issue is especially relevant for AI-assisted systems, whose performance may depend heavily on dataset composition, imaging conditions, and local population characteristics. For real-world implementation, diagnostic tools must demonstrate not only accuracy but also standardization, affordability, accessibility, and clear added value within existing clinical pathways [50,69,71,76,77].

For biomarker-based diagnostics, especially saliva- and blood-based assays, further challenges arise from pre-analytical and individual-level variability. In saliva studies, biomarker profiles may be influenced by circadian rhythm, diet, hydration, smoking, oral hygiene, periodontal inflammation, microbial contamination, and differences in collection or storage protocols. Blood- based assays face analogous limitations, including low tumor fraction, background cfDNA, and technical variability. Together, these factors contribute substantially to heterogeneity across studies and remain important barriers to the reproducible clinical implementation of biomarker-based early detection strategies [51,52,63,78]. Practical implementation also depends on factors that are rarely captured by diagnostic-accuracy metrics. Cost, equipment maintenance, chairside time, staff training, image interpretation, sample transport, laboratory turnaround time, reimbursement, patient acceptance, and local referral capacity may determine whether an adjunct improves care. A test that performs well in a specialist cohort may have limited benefit in general dental practice if it increases uncertain findings without accelerating biopsy or specialist review.

## 10. Future Directions

Future progress in early OSCC diagnosis is likely to depend on the integration of molecular biomarkers, non-invasive sampling strategies, advanced imaging, and computational analysis into more comprehensive diagnostic frameworks. Rather than replacing conventional histopathology, these emerging approaches are more likely to refine patient stratification, improve early lesion detection, and support longitudinal monitoring. Current developments suggest that the field is moving toward multimodal and data-integrated diagnostic models capable of capturing the biological complexity of oral carcinogenesis more effectively than any single biomarker or assay alone [51,63,71].

### 10.1. Multi-Omics Approaches

One of the most promising directions in oral cancer diagnostics is the increasing use of multi-omics strategies that integrate genomic, epigenomic, transcriptomic, proteomic, and metabolomic data. This approach is particularly attractive because oral carcinogenesis is biologically heterogeneous and cannot be fully characterized by isolated molecular markers. Multi-omics models may improve diagnostic sensitivity and specificity by capturing complementary layers of tumor biology, including mutational events, aberrant methylation, dysregulated RNA expression, altered protein profiles, and metabolic reprogramming [54,63].

In addition to enhancing early detection, such approaches may support longitudinal monitoring, risk stratification, and more precise characterization of disease progression. However, their successful implementation will depend on robust computational integration, standardized workflows, and independent validation across clinically relevant cohorts [64,78].

### 10.2. Artificial Intelligence

Artificial intelligence is expected to play an increasingly important role in the future of OSCC diagnostics, particularly in analyzing imaging data and complex molecular datasets. Machine learning algorithms, including random forest, support vector machine, artificial neural networks, and clustering-based models, have already been applied to oral images, radiological data, and molecular signatures to improve lesion detection, classification, and risk stratification.

Deep learning approaches, especially those based on convolutional neural networks, have shown particular promise in analyzing histopathologic images and clinical photographs, where they can identify subtle patterns that are not readily apparent to the human observer. In addition, AI-based integration of imaging, molecular, and clinical data may improve the interpretation of high-dimensional datasets and support more individualized diagnostic assessment [79,80,81].

Despite these advances, the current clinical validation level of most AI systems remains limited. Many models are trained and tested on retrospective, curated datasets rather than consecutive real-world patient cohorts, and performance may decline when applied to different cameras, staining protocols, clinical populations, image qualities, or disease prevalences. Dataset bias, class imbalance, unclear ground-truth annotation, inadequate external validation, and limited model explainability are major barriers to clinical adoption. AI outputs also require prospective assessment of whether they change management, reduce diagnostic delay, or improve referral and biopsy decisions rather than merely improving image-classification metrics.

For this reason, AI should currently be regarded as a decision-support research tool rather than a stand-alone diagnostic solution. Before routine use, models should undergo external validation, prospective multicenter testing, calibration in the intended care setting, assessment of interpretability and safety, and evaluation of workflow integration, including how AI outputs influence referral, biopsy, and follow-up decisions [69,71].

### 10.3. Biosensors

Biosensors represent another important area of development in early OSCC diagnostics because they offer the possibility of rapid, minimally invasive, and potentially point-of-care detection of tumor-associated molecules. These systems generally comprise a molecular recognition element, a signal transducer, and a signal-processing component, enabling the detection of proteins, DNA, RNA, or other cancer-related analytes in a quantitative or semi-quantitative manner.

Depending on the recognition system employed, biosensors may be protein-based, RNA-based, or DNA-based, each offering distinct advantages in terms of affinity, specificity, sensitivity, and molecular target range. In addition, biosensors may be classified according to their transduction mechanism, with electrochemical, optical, and nano-biosensor platforms being among the most frequently investigated in oral cancer research [70,72,73,74,75].

Among these approaches, electrochemical biosensors have attracted particular interest because of their relatively low cost, portability, rapid response, and high analytical sensitivity. Optical biosensors provide excellent specificity and real-time detection capabilities, but broader implementation may be limited by technical complexity and higher instrumentation costs. Nano-biosensors offer further opportunities for signal amplification and miniaturization, although issues related to stability, shelf life, and technical support remain important constraints.

Collectively, these technologies hold considerable promise for reducing patient discomfort, shortening diagnostic turnaround time, and facilitating decentralized or chairside testing. Nevertheless, their routine clinical use remains limited by challenges related to reproducibility, environmental stability, standardization, and large-scale validation [70,74,75].

Future biosensor development is therefore likely to focus on multimodal platforms capable of integrating multiple signal types, such as electrochemical, fluorescence-based, and surface plasmon resonance measurements, within a single device [74,75]. Additional advances may include improved sample handling, miniaturized electronics, wireless data transfer, and AI-assisted signal interpretation. If successfully validated, such systems may contribute to more accessible and clinically applicable diagnostic pathways for early OSCC detection and monitoring [70,72,82].

**Figure 2:**
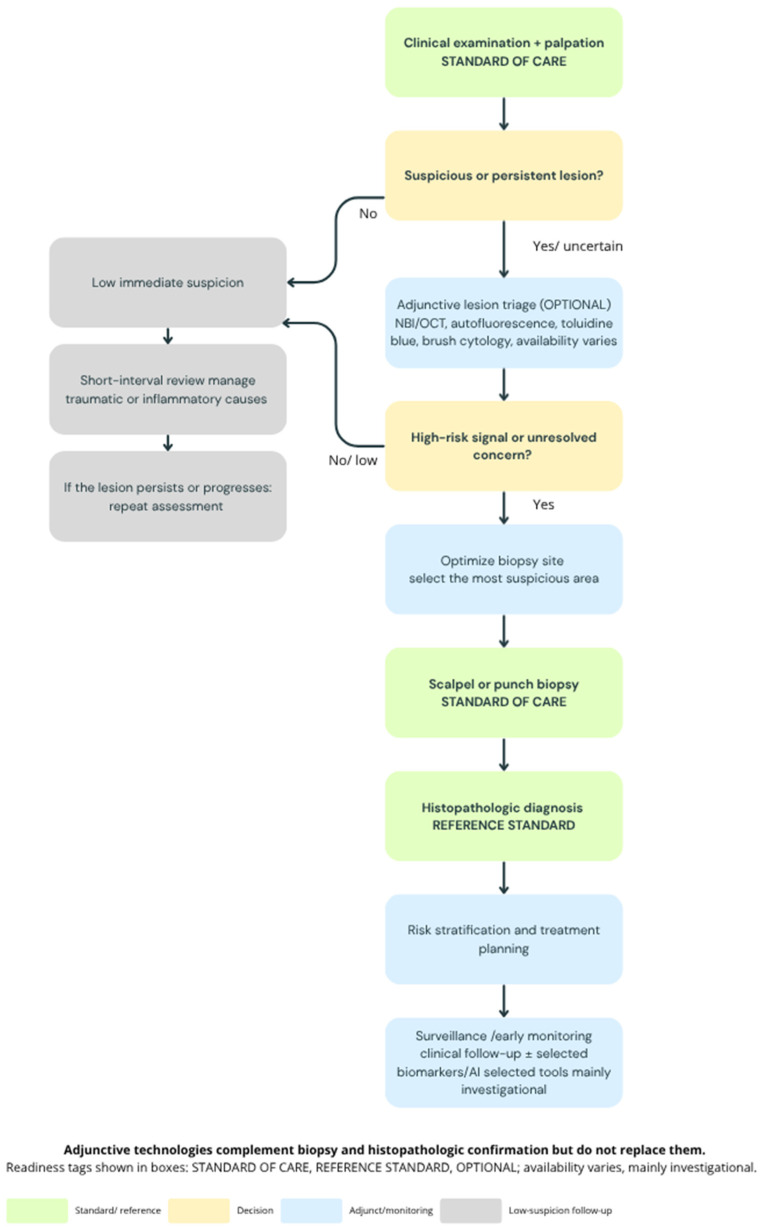
Proposed integrated pathway for early OSCC detection and early monitoring. Original figure created by the authors.

Taken together, these developments support a stepwise, integrated diagnostic pathway in which conventional clinical examination is followed by adjunctive lesion triage, biopsy-site optimization, histopathologic confirmation, risk stratification, and early surveillance using selected molecular and AI-assisted tools. The proposed pathway emphasizes that adjunctive technologies may refine clinical decision-making and monitoring, but should complement rather than replace biopsy and histopathological confirmation, as summarized in Figure 2.

## 11. Conclusions

Early diagnosis remains critical in OSCC, but the current clinical reality is that conventional examination, timely referral, and histopathologic confirmation remain the indispensable diagnostic foundation. No adjunctive imaging method, cytology platform, saliva-based biomarker, liquid-biopsy assay, AI model, or biosensor is currently validated sufficiently to replace biopsy for suspicious or persistent oral lesions.

NBI, OCT, and molecularly enhanced brush cytology appear to be among the better-supported adjuncts for lesion triage, biopsy guidance, or selected surveillance, particularly in specialist or risk-enriched settings. This statement should be interpreted as a relative assessment of translational readiness rather than a recommendation for routine population screening or unrestricted use in general dental practice.

Transition to routine clinical practice will require prospective multicenter validation in consecutive and clinically representative cohorts, predefined thresholds, standardized acquisition and interpretation protocols, external validation, assessment of cost and workflow impact, and evidence that the adjunct improves meaningful outcomes such as timely biopsy, reduced diagnostic delay, appropriate referral, or earlier recurrence detection. Until then, emerging biomarkers, AI, and biosensor platforms should be considered investigational or complementary decision-support tools.

## Figures and Tables

**Table 1:** Comparative characteristics of major liquid-biopsy analytes in early OSCC diagnosis [25,51,52,57,62].

Component	Target Analytes	Analytical Methods	Reported Diagnostic Relevance/Performance (Example Evidence)	Challenges
ctDNA and cfDNA	Somatic mutations, methylation, and fragmentomics	PCR-based methods, including ddPCR and BEAMing; NGS-based methods, including CAPP-Seq and TAM-Seq	In 123 HPV-negative OSCC patients, oral-rinse and plasma ctDNA detection rates were 94.3% and 80.5%, respectively; combined testing improved mutation detection sensitivity to 48.6% [52]	Detection limited to predefined mutations in select genes Low allele frequency Potential confounding by clonal hematopoiesis
CTCs	Intact tumor cells for phenotyping and genotyping characterization	CellSearch Assay (limited use)	Primarily prognostic/monitoring relevance; captures dissemination and EMT-related biology, but early-detection evidence in OSCC remains limited [51,57].	Low abundance in OSCC Technical complexity of capture and characterization
Exosomal and cell-free miRNAs	miRNAs, proteins, EV-associated DNA/RNA, and small non-coding RNAs	Microarray analysis and qRT-PCR	Eight-miRNA OC-vs-control model: AUC 0.954, sensitivity 86%, specificity 90% (OC n = 50; controls n = 60); four-miRNA OC-vs-OPMD model: AUC 0.9115, sensitivity 90%, specificity 82.7% (OPMD n = 52) [25].	Normalization issues Difficulty distinguishing host-derived from microbial vesicle-associated signals

**Table 2:** Comparative overview of current and emerging diagnostic modalities in early OSCC assessment [12,29,44,51,63,68,69,70].

Modality	Specimen/Input	Primary Clinical Application	Evidence Maturity/Selected Evidence Base	Main Limitation
Conventional oral examination	Direct visual inspection and palpation	Initial clinical assessment, identification of suspicious lesions, and referral decision-making	High; meta-analyses report sensitivity 63–71% and specificity 78–85% depending on inclusion criteria [11,27].	Limited sensitivity for subtle dysplasia or early invasion; operator dependence
Histopathologic biopsy	Tissue	Definitive diagnosis, grading, and margin assessment	High; diagnostic reference standard [28,29].	Invasive; subject to sampling error; unsuitable for repeated monitoring
Toluidine blue/vital staining	Oral mucosa	Biopsy-site selection, lesion triage, and adjunctive follow-up	Moderate; systematic review/meta-analysis evidence, but insufficient accuracy as a stand-alone test [47].	False positives in inflammatory or ulcerated lesions; false negatives in hyperkeratotic lesions
Brush cytology	Exfoliated epithelial cells	Minimally invasive lesion triage, early assessment, and repeat sampling	Moderate to high; PRISMA-DTA meta-analysis: 53 studies, 13,249 patients [44].	Misclassification risk; requires histopathologic confirmation
Cytology plus DNA ploidy/image cytometry	Brush sample/cellular material	Triage of high-grade lesions and risk enrichment	Moderate to high; prospective/algorithm studies suggest strong performance, but standardization and access vary [49].	Limited standardization; variable availability across centers
Autofluorescence	Optical tissue signal	Lesion triage, biopsy guidance, and follow-up of OPMDs	Moderate; light-based meta-analytic evidence, limited by false positives in benign/inflammatory lesions [27].	Reduced specificity in inflammatory and benign mucosal conditions
Chemiluminescence	Optical tissue signal	Lesion visualization and adjunctive triage	Low to moderate; pooled sensitivity 67% and specificity 48% in light-based meta-analysis [27].	Limited specificity and uncertain value for broad screening
Narrow-band imaging (NBI)	Optical vascular pattern	Lesion triage, vascular-pattern assessment, biopsy guidance, and selected surveillance	Moderate to high; OPMD/OSCC meta-analysis reports AUC 0.92 [39].	Requires expertise, equipment access, and standardized interpretation
Optical coherence tomography (OCT)	Cross-sectional optical imaging	Real-time architectural assessment, selected surgical or surveillance applications	Moderate to high; systematic review reports clinician-interpreted sensitivity ~75–92% and specificity ~71–93% [41].	Requires broader validation, cost, and workflow standardization
High-resolution microendoscopy/contact endoscopy	High-resolution optical imaging	Detailed evaluation of flagged lesions	Emerging; small specialist studies, with some evidence extrapolated from broader HNSCC/oral neoplasia settings [37,40].	Limited availability, specialist expertise, and modest real-world validation
Liquid biopsy: ctDNA/cfDNA	Saliva/oral rinse and blood	Molecular stratification, recurrence monitoring, and surveillance	Emerging; 123-patient HPV-negative OSCC dual-source ctDNA study [52].	Low tumor fraction, pre-analytical variability, and lack of harmonized cut-offs
Liquid biopsy: CTCs	Blood	Prognostication, assessment of dissemination biology, and monitoring	Emerging; limited OSCC-specific early detection evidence; stronger prognostic/monitoring rationale [51,57].	Low abundance in OSCC and technical capture complexity
Salivary miRNA/EV-based assays	Saliva	Early detection, risk stratification, and monitoring	Emerging; Balakittnen validation cohort: OC n = 50, OPMD n = 52, controls n = 60 [25].	Small cohorts, normalization issues, and limited external validation
Salivary proteomics/metabolomics	Saliva	Early detection, biological profiling, and risk enrichment	Emerging; exploratory systematic reviews and small/single metabolomics cohorts [63,64,65].	Biological confounding, pre-analytical variability, and reproducibility challenges
AI-assisted imaging/multimodal models	Images, optical data, and molecular data	Risk stratification, classification support, and decision support	Emerging; systematic reviews/meta-analyses, but external validation and workflow integration remain limited [69,71].	Dataset quality, external validation, interpretability, and workflow integration
Biosensor platforms	Saliva or other biofluids	Point-of-care testing and decentralized triage	Developmental to emerging; mostly proof-of-concept and review-level evidence [70,72,73,74,75].	Reproducibility, environmental stability, and large-scale clinical validation

## Data Availability

No new data were created or analyzed in this study. Data sharing is not applicable to this article.

## Abbreviations

The following abbreviations are used in this manuscript:

BEAMing	Beads, emulsion, amplification, and magnetics
CAPP-Seq	Cancer personalized profiling by deep sequencing
cfDNA	Cell-free DNA
CTCs	Circulating tumor cells
ctDNA	Circulating tumor DNA
ddPCR	Droplet digital PCR
EMT	Epithelial-to-mesenchymal transition
EVs	Extracellular vesicles
FNA	Fine-needle aspiration
HPV	Human papillomavirus
HRME	High-resolution microendoscopy
miRNA	microRNA
NGS	Next-generation sequencing
OCT	Optical coherence tomography
OPMDs	Oral potentially malignant disorders
OSCC	Oral squamous cell carcinoma
qRT-PCR	Quantitative reverse transcription polymerase chain reaction
SCNAs	Somatic copy number alterations
TAM-Seq	Tagged amplicon deep sequencing
UV	Ultraviolet
UVB	Ultraviolet B

## References

[1] Ferlay J., Ervik M., Lam F., Laversanne M., Colombet M., Mery L., Piñeros M., Znaor A., Soerjomataram I., Bray F. (2024). Global Cancer Observatory: Cancer Today.

[2] Tran Q., Maddineni S., Arnaud E.H., Divi V., Megwalu U.C., Topf M.C., Sunwoo J.B. (2023). Oral cavity cancer in young, non-smoking, and non-drinking patients: A contemporary review. Crit. Rev. Oncol..

[3] Stepan K.O., Mazul A.L., Larson J., Shah P., Jackson R.S., Pipkorn P., Kang S.Y., Puram S.V. (2023). Changing Epidemiology of Oral Cavity Cancer in the United States. Otolaryngol. Head Neck Surg..

[4] Stawarz K., Bieńkowska-Pluta K., Galazka A., Gorzelnik A., Durzynska M., Misiak-Galazka M., Stawarz G., Zwolinski J. (2025). Clinicopathologic Predictors of Survival Following Oral Cancer Surgery: A Retrospective Cohort Study. Cancers.

[5] Zanoni D.K., Montero P.H., Migliacci J.C., Shah J.P., Wong R.J., Ganly I., Patel S.G. (2019). Survival outcomes after treatment of cancer of the oral cavity (1985–2015). Oral Oncol..

[6] Evans M., Bonomo P., Chan P.C., Chua M.L.L., Eriksen J.G., Hunter K., Jones T.M., Laskar S.G., Maroldi R., O’Sullivan B. (2025). Post-operative radiotherapy for oral cavity squamous cell carcinoma: Review of the data guiding the selection and the delineation of post-operative target volumes. Radiother. Oncol..

[7] Rivera C. (2015). Essentials of oral cancer. Int. J. Clin. Exp. Pathol..

[8] Lingen M.W., Xiao W., Schmitt A., Jiang B., Pickard R., Kreinbrink P., Perez-Ordonez B., Jordan R.C., Gillison M.L. (2013). Low etiologic fraction for high-risk human papillomavirus in oral cavity squamous cell carcinomas. Oral Oncol..

[9] Chi A.C., Day T.A., Neville B.W. (2015). Oral cavity and oropharyngeal squamous cell carcinoma—An update. CA Cancer J. Clin..

[10] Chamoli A., Gosavi A.S., Shirwadkar U.P., Wangdale K.V., Behera S.K., Kurrey N.K., Kalia K., Mandoli A. (2021). Overview of oral cavity squamous cell carcinoma: Risk factors, mechanisms, and diagnostics. Oral Oncol..

[11] Essat M., Cooper K., Bessey A., Clowes M., Chilcott J.B., Hunter K.D. (2022). Diagnostic accuracy of conventional oral examination for detecting oral cavity cancer and potentially malignant disorders in patients with clinically evident oral lesions: Systematic review and meta-analysis. Head Neck.

[12] D’Orsi P.C.C., Warnakulasuriya S., Perri F., Monteiro L., Guida A. (2026). Beyond Visual Inspection: A Systematic Review of Adjunctive Aids for the Early Detection of Oral Squamous Cell Carcinoma. J. Clin. Med..

[13] Lau J., O G., Warnakulasuriya S., Balasubramaniam R., Frydrych A., Kujan O. (2024). Adjunctive aids for the detection of oral squamous cell carcinoma and oral potentially malignant disorders: A systematic review of systematic reviews. Jpn. Dent. Sci. Rev..

[14] Tatehara S., Satomura K. (2020). Non-invasive diagnostic system based on light for detecting early-stage oral cancer and high-risk precancerous lesions—Potential for dentistry. Cancers.

[15] Sowmya S.V., Augustine D., Haragannavar V.C., Khudhayr E.A. (2022). Cytogenetics in Oral Cancer: A Comprehensive Update. J. Contemp. Dent. Pract..

[16] Alshahrani S.A., Al-Qahtani W.S., Almufareh N.A., Domiaty D.M., Albasher G.I., Safhi F.A., AlQassim F.A., Alotaibi M.A., Al-Hazani T.M., Almutlaq B.A. (2021). Oral cancer among Khat users: Finding evidence from DNA analysis of nine cancer-related gene mutations. BMC Oral Health.

[17] Ali J., Sabiha B., Jan H.U., Haider S.A., Khan A.A., Ali S.S. (2017). Genetic etiology of oral cancer. Oral Oncol..

[18] Yong Z.W.E., Zaini Z.M., Kallarakkal T.G., Karen-Ng L.P., Rahman Z.A.A., Ismail S.M., Sharifah N.A., Mistafa W.M.W., Abraham M.T., Tay K.K. (2014). Genetic alterations of chromosome 8 genes in oral cancer. Sci. Rep..

[19] Tan Y., Wang Z., Xu M., Li B., Huang Z., Qin S., Nice E.C., Tang J., Huang C. (2023). Oral squamous cell carcinomas: State of the field and emerging directions. Int. J. Oral Sci..

[20] D’Mello S., Bavle R., Paremala K., Makarla S., Sudhakara M., Bhatt M. (2016). The synergy of tobacco and alcohol and glutathione S-transferase θ 1 gene deletion and oral squamous cell carcinoma. J. Oral Maxillofac. Pathol..

[21] Ardila C.M., Vivares-Builes A.M., Pineda-Vélez E. (2025). Molecular Biomarkers and Machine Learning in Oral Cancer: A Systematic Review and Meta-Analysis. Oral Dis..

[22] Gissi D.B., Rossi R., Lenzi J., Tarsitano A., Gabusi A., Balbi T., Montebugnoli L., Marchetti C., Foschini M.P., Morandi L. (2024). Thirteen-gene DNA methylation analysis of oral brushing samples: A potential surveillance tool for periodic monitoring of treated patients with oral cancer. Head Neck.

[23] Adeoye J., Alade A.A., Zhu W., Wang W., Choi S.-W., Thomson P. (2022). Efficacy of hypermethylated DNA biomarkers in saliva and oral swabs for oral cancer diagnosis: Systematic review and meta-analysis. Oral Dis..

[24] Rapado-González Ó., Salta S., López-López R., Henrique R., Suárez-Cunqueiro M.M., Jerónimo C. (2024). DNA methylation markers for oral cancer detection in non- and minimally invasive samples: A systematic review. Clin. Epigenetics.

[25] Balakittnen J., Weeramange C.E., Wallace D.F., Duijf P.H.G., Cristino A.S., Hartel G., Barrero R.A., Taheri T., Kenny L., Vasani S. (2024). A novel saliva-based miRNA profile to diagnose and predict oral cancer. Int. J. Oral Sci..

[26] Liu S., Li J., Shao Q., Chen J., Zou C., Ai Y. (2025). Uncovering biomarkers and pathways in oral squamous cell carcinoma through integrated lncRNA-mRNA regulatory network analysis. Discov. Oncol..

[27] Buenahora M.R., Peraza L.A., Díaz-Báez D., Bustillo J., Santacruz I., Trujillo T.G., Lafaurie G.I., Chambrone L. (2021). Diagnostic accuracy of clinical visualization and light-based tests in precancerous and cancerous lesions of the oral cavity and oropharynx: A systematic review and meta-analysis. Clin. Oral Investig..

[28] Poh C.F., Ng S., Berean K.W., Williams P.M., Rosin M.P., Zhang L. (2008). Biopsy and Histopathologic Diagnosis of Oral Premalignant and Malignant Lesions. Can. Dent. Assoc..

[29] Urquhart O., Bhosale A.S., Martins-Pfeifer C., Verdugo-Paiva F., Carrasco-Labra A., Pimentel J., Sadek N., Agrawal N., Chaturvedi A.K., Gurenlian J. (2026). Living evidence-informed guideline on the early detection of oral squamous cell carcinoma and potentially malignant disorders: Cytology adjuncts to determine the need for biopsy, Version 2026 1.0. J. Am. Dent. Assoc..

[30] Tenore G., Mohsen A., Nuvoli A., Palaia G., Rocchetti F., Di Gioia C.R.T., Cicconetti A., Romeo U., Del Vecchio A. (2023). The Impact of Laser Thermal Effect on Histological Evaluation of Oral Soft Tissue Biopsy: Systematic Review. Dent. J..

[31] Meemongkol N., Jantharapattana K., Thongsuksai P. (2023). Quantitative and qualitative features of tissue biopsy for successful diagnosis of oral cancer. Oral Oncol. Rep..

[32] Yang G., Wei L., Thong B.K.S., Fu Y., Cheong I.H., Kozlakidis Z., Li X., Wang H., Li X. (2022). A Systematic Review of Oral Biopsies, Sample Types, and Detection Techniques Applied in Relation to Oral Cancer Detection. BioTech.

[33] Pynnonen M.A., Gillespie M.B., Roman B., Rosenfeld R.M., Tunkel D.E., Bontempo L., Brook I., Chick D.A., Colandrea M., Finestone S.A. (2017). Clinical Practice Guideline: Evaluation of the Neck Mass in Adults. Otolaryngol. Head Neck Surg..

[34] Saka-Herrán C., Jané-Salas E., Mari-Roig A., Estrugo-Devesa A., López-López J. (2021). Time-to-treatment in oral cancer: Causes and implications for survival. Cancers.

[35] Lingen M.W., Tampi M.P., Urquhart O., Abt E., Agrawal N., Chaturvedi A.K., Cohen E., D’souza G., Gurenlian J., Kalmar J.R. (2017). Adjuncts for the evaluation of potentially malignant disorders in the oral cavity: Diagnostic test accuracy systematic review and meta-analysis—A report of the American Dental Association. Am. Dent. Assoc..

[36] Fedele S. (2009). Diagnostic aids in the screening of oral cancer. Head Neck Oncol..

[37] Muldoon T.J., Roblyer D., Williams M.D., Stepanek V.M.T., Richards R., Gillenwater A.M. (2012). Noninvasive imaging of oral neoplasia with a high-resolution fiber-optic microendoscope. Head Neck.

[38] Rashid A., Warnakulasuriya S. (2015). The use of light-based (optical) detection systems as adjuncts in the detection of oral cancer and oral potentially malignant disorders: A systematic review. J. Oral Pathol. Med..

[39] van Schaik J.E., Halmos G.B., Witjes M.J.H., Plaat B.E.C. (2021). An overview of the current clinical status of optical imaging in head and neck cancer with a focus on Narrow Band imaging and fluorescence optical imaging. Oral Oncol..

[40] Pošta P., Kolk A., Pivovarčíková K., Liška J., Genčur J., Moztarzadeh O., Micopulos C., Pěnkava A., Frolo M., Bissinger O. (2023). Clinical Experience with Autofluorescence Guided Oral Squamous Cell Carcinoma Surgery. Diagnostics.

[41] Jerjes W., Stevenson H., Ramsay D., Hamdoon Z. (2024). Enhancing Oral Cancer Detection: A Systematic Review of the Diagnostic Accuracy and Future Integration of Optical Coherence Tomography with Artificial Intelligence. J. Clin. Med..

[42] Perdiou A., Dumitrescu R., Jumanca D., Balean O., Sava-Rosianu R., Talpos S., Lalescu D.V., Galuscan A. (2025). Leveraging Autofluorescence for Tumor Detection, Diagnosis, and Accurate Excision with Surgical Margin Assessment in Tumor Excision. Dent. J..

[43] Wang P., Wang S., Zhang Y., Duan X. (2022). Multispectral Image under Tissue Classification Algorithm in Screening of Cervical Cancer. J. Healthc. Eng..

[44] Tayebi-Hillali H., Lorenzo-Pouso A.I., Marichalar-Mendía X., Gándara-Vila P., Reboiras-López D., Blanco-Carrión A., Coppini M., Caponio V.C.A., Pérez-Sayáns M. (2025). Accuracy of Cytological Methods in Early Detection of Oral Squamous Cell Carcinoma and Potentially Malignant Disorders: A Systematic Review and Meta-Analysis. J. Oral Pathol. Med..

[45] Sukegawa S., Ono S., Nakano K., Takabatake K., Kawai H., Nagatsuka H., Furuki Y. (2020). Clinical study on primary screening of oral cancer and precancerous lesions by oral cytology. Diagn. Pathol..

[46] Vijayakumar V., Reghunathan D., Edacheriyan B., Mukundan A. (2019). Role of Toluidine Blue Staining in Suspicious Lesions of Oral Cavity and Oropharynx. Indian J. Otolaryngol. Head Neck Surg..

[47] Kim D.H., Song E.A., Kim S.W., Hwang S.H. (2021). Efficacy of toluidine blue in the diagnosis and screening of oral cancer and pre-cancer: A systematic review and meta-analysis. Clin. Otolaryngol..

[48] McRae M.P., Modak S.S., Simmons G.W., Trochesset D.A., Kerr A.R., Thornhill M.H., Redding S.W., Vigneswaran N., Kang S.K., Christodoulides N.J. (2020). Point-of-care oral cytology tool for the screening and assessment of potentially malignant oral lesions. Cancer Cytopathol..

[49] Bechstedt N., Pomjanski N., Schramm M., Remmerbach T.W. (2022). Evaluation of Static DNA Ploidy Analysis Using Conventional Brush Biopsy-Based Cytology Samples as an Adjuvant Diagnostic Tool for the Detection of a Malignant Transformation in Potentially Oral Malignant Diseases: A Prospective Study. Cancers.

[50] Balapure A., Dubey S.K., Javed A., Chattopadhyay S., Goel S. (2024). A review: Early detection of oral cancer biomarkers using microfluidic colorimetric point-of-care devices. Anal. Methods.

[51] Niekra P., Adamska P. (2026). The Role of Liquid Biopsy in the Diagnosis of Oral Squamous Cell Carcinoma: A Systematic Review. Int. J. Mol. Sci..

[52] Chen Z., Chen L., Li S., Xia P., Lam A.K.-Y., Qiao J., Liu Y., Qiao B. (2025). Integrated analysis of oral rinse-derived and plasma circulating tumour DNA for mutation profiling and outcome prediction with oral squamous cell carcinoma. NPJ Precis. Oncol..

[53] Naito Y., Honda K. (2023). Liquid Biopsy for Oral Cancer Diagnosis: Recent Advances and Challenges. J. Pers. Med..

[54] Esperouz F., Ciavarella D., Santarelli A., Lorusso M., Muzio L.L., Laino L., Russo L.L. (2024). Saliva-Based Biomarkers in Oral Squamous Cell Carcinoma Using OMICS Technologies: A Systematic Review. Oral.

[55] Rapado-González Ó., Rodríguez-Ces A.M., López-López R., Suárez-Cunqueiro M.M. (2023). Liquid biopsies based on cell-free DNA as a potential biomarker in head and neck cancer. Jpn. Dent. Sci. Rev..

[56] Rigotti P., Polizzi A., Quinzi V., Blasi A., Lombardi T., Muzio E.L., Isola G. (2025). Cell-Free DNA as a Prognostic Biomarker in Oral Carcinogenesis and Oral Squamous Cell Carcinoma: A Translational Perspective. Cancers.

[57] Wikner J., Gröbe A., Pantel K., Riethdorf S. (2014). Squamous cell carcinoma of the oral cavity and circulating tumour cells. World J. Clin. Oncol..

[58] Osan C., Berindan-Neagoe I., Dinu C., Armencea G., Bran S., Kretschmer W., Baciut G., Onisor F., Baciut M. (2025). Salivary MicroRNAs as Innovative Biomarkers for Diagnosis and Prediction of the Oral Potentially Malignant Disorders Transition Towards Oral Cancer: A Systematic Review. J. Clin. Med..

[59] Qin Y., Dong X., Li B. (2025). Salivary miRNAs and cytokines associated with diagnosis and prognosis of oral squamous cell carcinoma. Front. Cell Dev. Biol..

[60] Owecki W., Wojtowicz K., Nijakowski K. (2025). Salivary Extracellular Vesicles in Detection of Head and Neck Cancers: A Systematic Review. Int. J. Nanomed..

[61] Sanesi L., Mori G., Troiano G., Ballini A., Valzano F., Dioguardi M., Muzio L.L., Magalhaes M., Caponio V.C.A. (2024). Salivary exosomal microRNA profile as biomonitoring tool for diagnosis and prognosis of patients with head and neck squamous cell carcinoma: A systematic review. Arch. Oral Biol..

[62] Deorah S., Singh A., Gupta S. (2024). Beyond tissue: Liquid biopsy’s promise in unmasking oral cancer. Oral Oncol. Rep..

[63] Bastías D., Maturana A., Marín C., Martínez R., Niklander S.E. (2024). Salivary Biomarkers for Oral Cancer Detection: An Exploratory Systematic Review. Int. J. Mol. Sci..

[64] Antonelli R., Setti G., Treister N.S., Pertinhez T.A., Ferrari E., Gallo M., Bologna-Molina R., Vescovi P., Meleti M. (2024). Salivary metabolomics in oral cancer: A systematic review. Oral Oncol. Rep..

[65] Panneerselvam K., Krishnan R., Mahadevan S., Ayyadurai M.M., Sugimoto M. (2026). Salivary monoacetylated polyamines as noninvasive biomarkers for early detection and stratification of oral squamous cell carcinoma: A targeted metabolomics study. Arch. Oral Biol..

[66] Hu Y., Xu M., Liu M., Peng H. (2025). Comparison of saliva and blood derived cell free RNAs for detecting oral squamous cell carcinoma. Sci. Rep..

[67] Constantin V., Luchian I., Goriuc A., Budala D.G., Bida F.C., Cojocaru C., Butnaru O.-M., Virvescu D.I. (2025). Salivary Biomarkers Identification: Advances in Standard and Emerging Technologies. Oral.

[68] Gürhan C., İlhan B., Gürses B.O., Bölükbaşı G., Güneri P. (2025). The influence of toluidine blue staining on decision-making for the selection of biopsy sites in oral disorders. Eur. Oral Res..

[69] Horvath D., Fekete A., Martinekova P., Kiss-Dala S., Abram E., Marton K., Zsembery A., Hegyi P., Brody A. (2026). Artificial Intelligence Helps Diagnose Oral Potentially Malignant Disorders: A Systematic Review and Meta-Analysis. JDR Clin. Trans. Res..

[70] Nagdeve S.N., Suganthan B., Ramasamy R.P. (2025). Perspectives on the Application of Biosensors for the Early Detection of Oral Cancer. Sensors.

[71] Sahoo R.K., Sahoo K.C., Dash G.C., Kumar G., Baliarsingh S.K., Panda B., Pati S. (2024). Diagnostic performance of artificial intelligence in detecting oral potentially malignant disorders and oral cancer using medical diagnostic imaging: A systematic review and meta-analysis. Front. Oral Heal..

[72] Zhu X., Chen J., Zhao J., Zhang Z., Wang C. (2025). Nanomaterials for Theranostic Management of Oral Cancer: Advances in Imaging, Biosensing, Targeted Delivery, and Multimodal Synergistic Therapy. Int. J. Nanomed..

[73] Umapathy V.R., Natarajan P.M., Swamikannu B., Moses J., Jones S., Chandran M.P., Anbumozhi M.K. (2022). Emerging Biosensors for Oral Cancer Detection and Diagnosis—A Review Unravelling Their Role in Past and Present Advancements in the Field of Early Diagnosis. Biosensors.

[74] Teow J.Y., Zhang Q., Abidin S.A.Z., Tan C.C., Rahman S.N.S.A., Karsani S.A., Othman I., Chen Y., Lakshmipriya T., Gopinath S.C. (2024). Pioneering biosensor approaches for oral squamous cell carcinoma diagnosis: A comprehensive review. Process Biochem..

[75] Goldoni R., Scolaro A., Boccalari E., Dolci C., Scarano A., Inchingolo F., Ravazzani P., Muti P., Tartaglia G. (2021). Malignancies and biosensors: A focus on oral cancer detection through salivary biomarkers. Biosensors.

[76] Mansukhbhai T.V., Patidar M., Chakor M., Patil D.V., Rebello P., Mohanty G., Laddha R. (2025). Potential use of liquid biopsy in diagnosing oral malignancies. Bioinformation.

[77] Prasad M., Sekar R., Priya M.D.L., Varma S.R., Karobari M.I. (2024). A new perspective on diagnostic strategies concerning the potential of saliva-based miRNA signatures in oral cancer. Diagn. Pathol..

[78] Starska-Kowarska K. (2025). Salivaomic Biomarkers—An Innovative Approach to the Diagnosis, Treatment, and Prognosis of Oral Cancer. Biology.

[79] Saraswathi T., Bhaskaran D.V.M. (2026). An effective oral cancer detection, risk stratification analysis, and survival prediction framework using ROI segmentation with generative AI techniques. Biomed. Signal Process. Control.

[80] Liu-Swetz Y., Niksic S., Seethala R.R., Shasteen A., Foran D.J., Bilodeau E.A. (2025). AI-driven prediction of progression to oral squamous cell carcinoma using a multiresolution pathology model. NPJ Digit. Med..

[81] Dixit S., Kumar A., Srinivasan K. (2023). A Current Review of Machine Learning and Deep Learning Models in Oral Cancer Diagnosis: Recent Technologies, Open Challenges, and Future Research Directions. Diagnostics.

[82] Tai Y., Li Y., Mornay K.M., Woodard M.K., Wang W., Yang X., Pan J. (2026). Biosensors in dental, oral and craniofacial applications. Npj Biosensing.

